# Microparticle Transport and Sedimentation in a Rhythmically Expanding Alveolar Chip

**DOI:** 10.3390/mi13030485

**Published:** 2022-03-20

**Authors:** Wei Zhang, Jun Dong, Huimin Lv, Weitao Bai, Hongzhou Lu, Bernd R. Noack, Yonggang Zhu, Yue Yang

**Affiliations:** 1 School of Mechanical Engineering and Automation, Harbin Institute of Technology, Shenzhen 518055, China; zhang.wei2022@foxmail.com (W.Z.); d.j.dong@foxmail.com (J.D.); huimin2019@foxmail.com (H.L.); hitszwt@163.com (W.B.); bernd.noack@hit.edu.cn (B.R.N.); zhuyonggang@hit.edu.cn (Y.Z.); 2 National Clinical Research Centre for Infectious Diseases, The Third People’s Hospital of Shenzhen (The Second Affiliated Hospital of Southern University of Science and Technology), Shenzhen 518112, China; luhongzhou@szsy.sustech.edu.cn

**Keywords:** alveolar chip, particle tracking, microfluidics, dynamical similarity, particle deposition

## Abstract

Understanding the mechanism of particle transport and sedimentation in pulmonary alveolus is important for deciphering the causes of respiratory diseases and helping the development of drug delivery. In this study, taking advantage of the microfluidic technique, an experimental platform was developed to study particle behavior in a rhythmically expanding alveolar chip for a sufficient number of cycles. The alveolar flow patterns at different generations were measured for two cases with the gravity direction parallel or vertical to the alveolar duct. Affected by both the vortex flow inside the alveoli and the shear flow in the duct simultaneously, it was observed that particles inside the alveoli either escaped from the inlet of the alveolar duct or stayed in the alveoli, revealing the irreversibility of particle transport in the alveoli. At the earlier acinar generations, particles were inclined to deposit on the distal alveolar wall. The settling rates of particles of different sizes in the alveoli were also compared. This study provides valuable data for understanding particle transport and sedimentation in the alveoli.

## 1. Introduction

Microparticles or toxic aerosol reaching the pulmonary acinar regions by human respiration can lead to many lung diseases such as chronic obstructive pulmonary disease (COPD), lung cancer, etc. [1,2,3,4]. The effective delivery of aerosol-based drugs through human breathing into the lungs is currently receiving great attention [5,6,7,8]. Understanding the mechanism of particle transport and sedimentation in pulmonary alveoli is of great significance in deciphering the causes of respiratory diseases and helping the development of inhalation therapy. Due to the difficulty of mimicking the realistic and dynamic respiration process, and tracking particle motion for a sufficient number of cycles, most research on particle transport and sedimentation in the acinar region is still reliant on numerical simulations [8,9,10,11,12,13,14,15,16].

Several experiments have been carried out to study particle transport and sedimentation in the alveoli, including scaled-up models and microfluidic chips [17,18,19,20,21,22,23,24,25]. Chhabra et al. [26] and Oakes et al. [17] developed scaled-up alveolar models and adopted the particle imaging velocimetry (PIV) system to investigate particle transport and deposition in the alveoli. Rong et al. [18,25] studied the effect of functional residual capacity on the deposition of particles in the human alveolar region by setting up a scale-up model with multiple alveoli. However, it is hard to achieve the dynamic similarity of particle transport between the human alveolar flow and the scale-up models. Compared to the scale-up models, microfluidic chips provided the possibility of studying the behavior of particle transport in the alveoli on a real scale. Sznitman’s group [19,20,22] developed a microfluidic platform with 5-generation alveoli and achieved cyclic alveolar expansion and contraction. A mixture solution of glycerol/water was used as the working fluid to achieve the hydrodynamic characteristics of the alveolar environment. Fishler et al. [20] tracked smoke particles mixed in the air in the 5-generation alveolar chip in one breathing cycle. The density of these particles was so great that they deposited on the alveolar wall quickly. The polystyrene microspheres mixed in the glycerol/water solution were tracked in the alveoli and alveolar ducts for several breathing cycles in the subsequent work by Fishler et al. [22]. However, the dynamic characteristics of the particles’ transport in the alveoli were not considered, and the trajectories of the polystyrene microspheres were similar to those of massless tracers.

Tsuda et al. [27] predicted that there existed chaotic flow in the alveolar flow through numerical simulations in 1995. Recently, Lv et al. [28] experimentally observed the existence of stagnation saddle points in the alveoli with a microfluidic chip, providing strong evidence to verify Tsuda’s prediction. Understanding the intricate behavior of microparticle transport in the chaotic flow of the alveoli is of great significance [29,30]. Dong et al. [31] revealed the trajectories of silver particles mixed in the glycerol/water solution in the alveoli under gravity with different directions. However, the particles were tracked for only a few breathing cycles, and it is difficult to discover the characteristics of particle transport and deposition in the alveoli. Tsuda et al. [32] showed that the average diameter of the inhalable particles is about 5.3 μm. Some researchers noticed that ultrafine particles with a diameter no more than 0.1 μm could enter deeper into the lungs than PM2.5 does [33,34,35,36]. Therefore, it is also important to experimentally investigate particle transport and sedimentation in the alveoli with different sizes.

The aim of this paper is to study transport of particles of different sizes in a microfluidic alveolar chip for a sufficient number of cycles. In addition, two cases with the gravity direction parallel or vertical to the alveolar duct direction are considered to represent the real alveoli located at different positions in the human body. The dynamic similarity of particle transport in the alveolar chip compared to the real case was guaranteed by matching multiple dimensionless parameters, which account for the effects of particle gravity, the drag force from the fluid, and particle diffusion. By tracking particle transport for a sufficient number of cycles, we were able to detect in detail the characteristics of particles escaping from the alveolus and particle transport in the alveolus at different generations.

## 2. Materials and Methods

### 2.1. Experimental System

**Figure 1:**
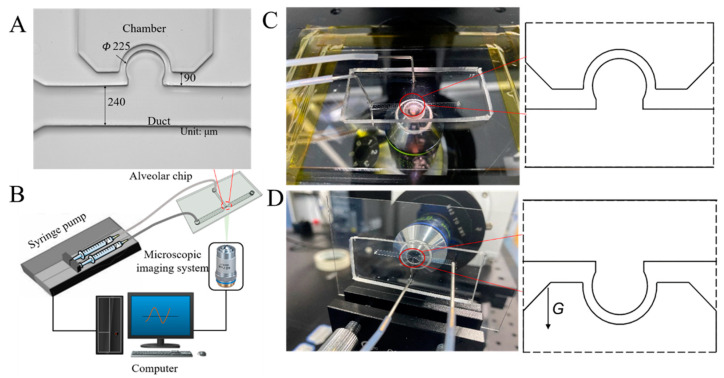
The alveolus chip and experimental system. (**A**) Photograph of the alveolar chip. (**B**) Schematic diagram of experimental device. (**C**) Alveolar chip placed horizontally. (**D**) Alveolar chip placed vertically.

The experimental system consisted of an alveolar chip, a pump system, and a microscopic imaging system, as shown in Figure 1A,B. The alveolar chip was placed in two directions to study the gravity effect, one perpendicular to the direction of gravity (Figure 1C) and the other in the direction of gravity (Figure 1D). A standard soft-lithography was used for fabricating the alveolar chip, and the mold of the alveolar chip was cast with a mixture of polydimethylsiloxane (PDMS)/curing agent at 10:1 (*w*/*w*) [19,28]. The alveolar chip was mainly composed of a cuboid alveolar duct with a square cross-section (240 μm in side length) lined with a cylindrical alveolus (225 μm in diameter) and a pressure chamber with a rectangular cross-section, and the thickness of the alveolar ductal wall was 90 μm (see Figure 1A). The alveolar chip had a quasi-three-dimensional structure, similar to in previous research work [28,31].

The pump system mainly included two syringe pumps (CETONI), two microinjectors, steel needles, and plastic conduits. The two syringe pumps were programmed to mimic a quiet breathing in sinusoidal mode, and the breathing period (*T*) was 4s. One syringe pump was connected to a 50 μL microinjector for controlling the mixer solution with particles in the alveolar duct. The other syringe pump was connected to a 10 mL or 25 mL injector to control the pressure inside the air chamber for the expansion and contraction of the alveolus.

The microscopic imaging system mainly consisted of a Photometrics high-speed camera Phantom VEO 710L (pixels) with a 40× objective and a computer. There were two kinds of experimental platform because of the different ways of placing the alveolar chip. When the alveolar chip was placed horizontally, the high-speed camera was mounted on the microscope (IX73, Olympus, Tokyo, Japan), as shown in Figure 1C. When the chip was placed vertically, the high-speed camera was fixed on a small anti-vibration platform, and the chip was fixed on the displacement platform (Figure 1D), which was similar to the previous published work [31]. Image-pro plus 6.0 was used for particle tracking in image post-processing.

### 2.2. Flow Visualization

To achieve the dynamic similarity of the alveolar flow, two dimensionless parameters were considered (Reynolds number *Re* and Wormsley number *Wo*).

(1) $$Re=\frac{uD}{\nu }$$

(2) $$Wo=D\sqrt{\frac{\omega }{\nu }}$$

where *u* is the flow velocity in the alveolar duct, *D* is the hydraulic diameter of the alveolar duct, *ν* is the kinematic viscosity, and *ω* is the pulsating angular frequency of flow in the alveolar duct, *ω* = 2*π*/*T*.

The mixture solution of glycerol/water with a mass ratio of 68:32 was chosen as the substitute for air in human alveoli. The kinematic viscosity and the density of air at 37 °C were about 1.67 × 10^−5^ m^2^/s and 1.11 kg/m^3^, respectively. The kinematic viscosity and the density of the mixture solution at 20 °C were 1.65 × 10^−5^ m^2^/s and 1.18 kg/m^3^, respectively. The detailed flow parameters and dimensions of the alveoli at different generations (from 15th to 23rd generation) were similar to Lv, et al. [28].

The alveolar flow was visualized by means of tracer microparticles moving with the streaming flow when the alveolar chip was placed vertically. The polystyrene microspheres (diameter *d*_p_ = 2 μm, density *ρ*_p_ = 1.05 g/cm^3^) were used as tracer particles. The momentum relaxation time $\tau_{p}=\frac{\rho_{p}d_{p}^{2}}{18\mu }$ (*μ* is the dynamic viscosity) was calculated, and $\tau_{p}=0.013\;\mu s$, which was very small relative to the timescale of fluid velocity changes, and it can be considered that the particle trajectories were identical to the movement of the carrier fluid [23]. The frame rates of the high-speed camera were 250, 200, 100, 70, 50, 30, 25, and 25 fps from the 16th to 23rd acinar generations, respectively. Tracer trajectories of the alveolar flow could be visualized by the superposition of a series of consecutive images.

### 2.3. Dynamic Similarity of Particle Transport

To achieve the dynamic similarity of the particle transport between the alveolar chip and the human alveoli, three dimensionless parameters were considered (Stokes number *Stk*, Gravity number *H*, and particle Peclet number *Pe*_p_). The Stokes number characterizes the importance of fine particle inertia in the fluid. The Gravity number characterizes the gravitational sedimentation when particles transport in the fluid. The particle Peclet number characterizes the ratio of the effect of convection to diffusion on particle transport in the fluid [11,31]. *Stk*, *H*, and *Pe*_p_ can be defined as

(3) $$Stk=\frac{\omega \rho_{p}d_{p}^{2}C_{c}}{18\mu }$$

(4) $$H=\frac{g\rho_{p}d_{p}^{2}C_{c}}{9\mu D_{d}\omega }$$

(5) $$Pe_{p}=\frac{3\pi D_{d}^{2}\omega \mu d_{p}}{k_{B}T_{\mathrm{em}}C_{c}}$$

where *g* is the gravitational acceleration, *k*_B_ is Boltzmann’s constant, and $k_{B}=1.38\times 10^{-23}\;J/K$; *T*_em_ is the temperature in Kelvin, and *C*_c_ is the Cunningham slip correction factor; when $d_{p}>0.1\;\mu m$, considering $C_{c}=1+2.52\lambda /d_{p}$, where λ is the mean free path of molecules in the fluid. For air, at room temperature and standard atmospheric pressure, λ is 0.067 μm. The mean free path of liquid molecules is smaller than the gap between adjacent molecules, so the mean free path of the mixture solution of the glycerol/water is ignored here.

**Figure 2:**
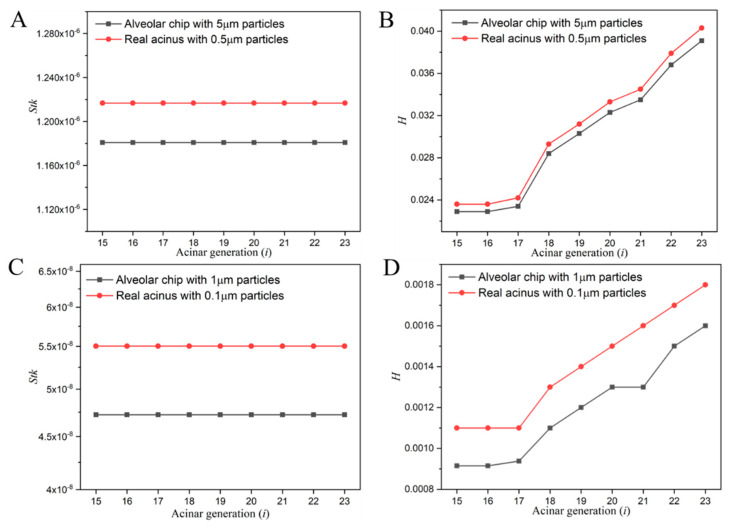
Comparison of dimensionless particle number at different acinar generations. (**A**) Stokes number of 5 μm silver particle. (**B**) Gravity number of 5 μm silver particle. (**C**) Stokes number of 1 μm silver particle. (**D**) Gravity number of 1 μm silver particle.

To match the dimensionless flow parameters, the spherical silver particles (density $\rho_{p}=10.5{\;g/\mathrm{cm}}^{3}$) with a diameter of 5 μm and 1 μm were chosen to represent the inhaled particles (density $\rho_{p}=1.0{\;g/\mathrm{cm}}^{3}$) with a diameter of 0.5 μm and 0.1 μm in the case of the real human respiratory process, respectively. The dimensionless numbers of particles in the alveolar chip and in the human alveoli were calculated and compared, as shown in Figure 2. The relative differences in the *Stk* and *H* of particles in the alveolar chip between the present model and real acinus at different generations were all within 20%, which is acceptable considering so many dimensionless parameters were being matched at the same time.

## 3. Results and Discussion

### 3.1. Alveolar Chip Placed Horizontally

**Figure 3:**
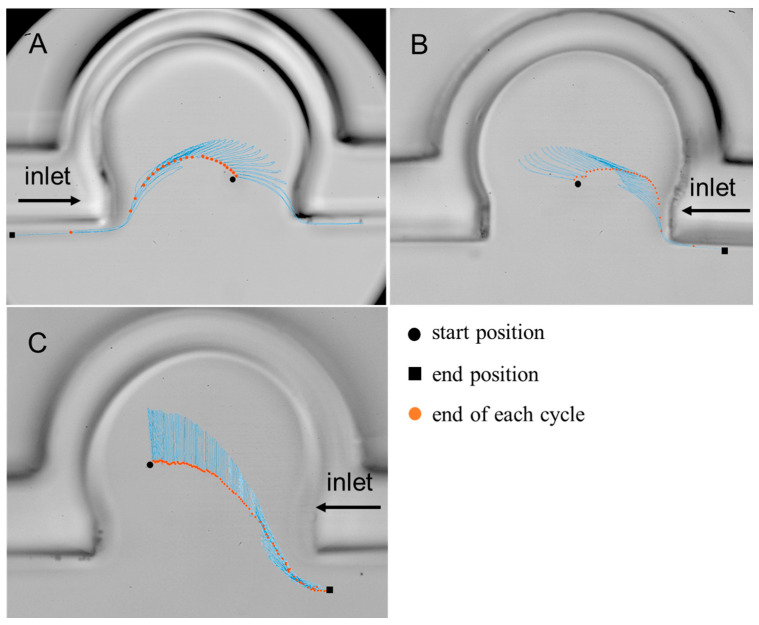
Trajectories of particles in horizontally placed chip. (**A**) A 5 μm particle over 24 *T* at the 21st generation. (**B**) A 1 μm particle over 34 *T* at the 21st generation. (**C**) A 1 μm particle over 101 *T* at the 23rd generation.

Figure 3 shows trajectories of particles with different sizes at the 21st and 23rd acinar generation in the horizontally placed chip. The high-speed camera with a frame rate of 25 fps was applied to take photos of silver particle positions for a sufficient number of breathing cycles in the experiment. Then, we determined these silver particle positions from image analysis and plotted the real particle trajectories. We tracked the particles for a long time, so the particle trajectories displayed on the finite-size graph are visually overlapping. The blue lines represent the particle trajectories. The black dots represent the particle start positions and the black squares represent the particle end positions. The orange dots are the particle positions at end of each breathing cycle. There was a vortex and a radial flow coexisting at the 21st acinar generation, and there was only radial flow at the 23rd acinar generation [27,28]. The start positions of particles were all inside the single alveolus. As the human breathing cycles increased to a certain number, the particles finally escaped from the alveoli and entered the alveolar duct. In one breathing cycle, the trajectories of particles in the exhalation were similar to that in the inhalation, but they did not overlap exactly, reflecting the irreversibility of particle trajectories. The particle positions were very close to the original positions after one breathing cycle. It was found that particles in the alveoli either escaped from the inlet side of the alveolar duct or stayed in the alveoli after sufficient breathing cycles. The behavior was independent of the direction of the inlet and the size of particles (Figure 3B,C). This phenomenon revealed that particle transport in alveoli is irreversible.

### 3.2. Alveolar Chip Placed Vertically

#### 3.2.1. Instantaneous Flow Patterns in Alveoli

**Figure 4:**
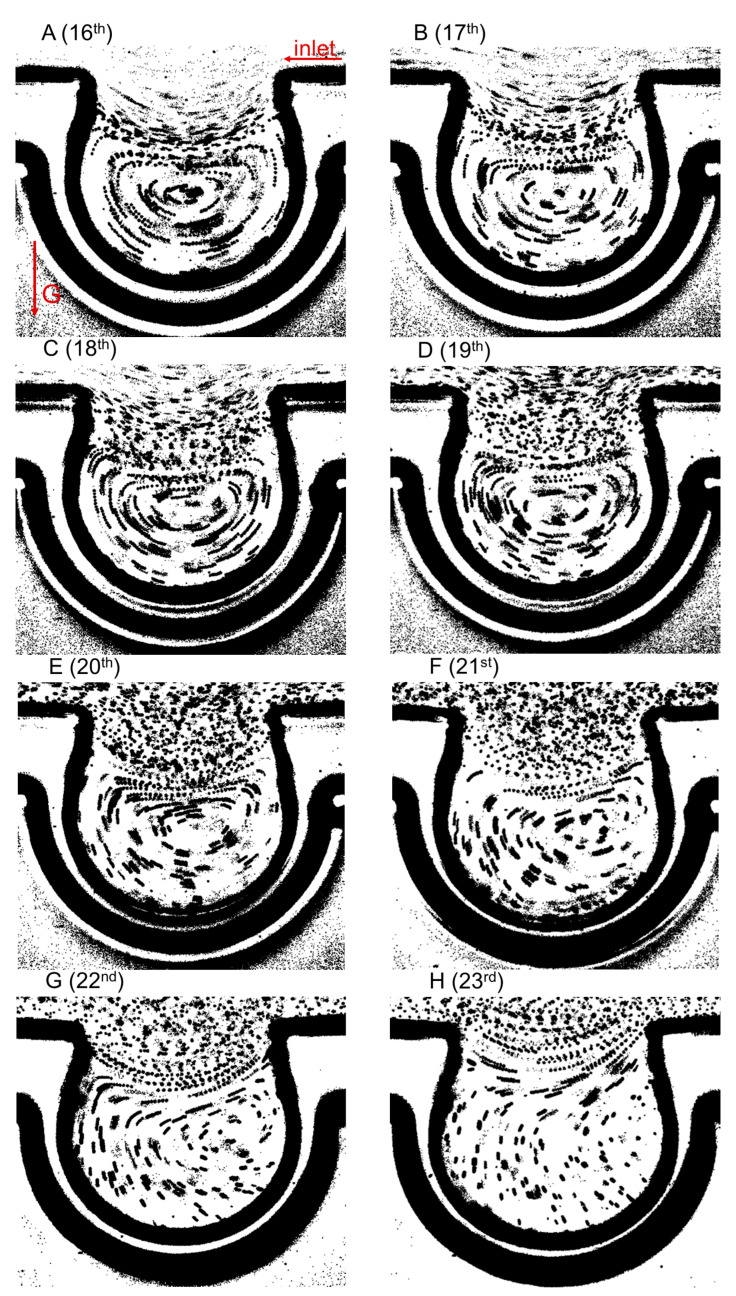
Visualized flow fields at *t* = *T*/4 (peak of inhalation) in the vertically placed chip. (**A**–**H**) are the measurements for the alveoli at the 16th to 23rd acinar generations, respectively.

The alveolar flow patterns at different acinar generations in vertically placed single alveolar chip were measured by superimposing eight subsequent single snapshots, as shown in Figure 4. The ductal inlet was on the right of the alveolus, and the gravitational direction was opposite to the direction of the alveolar opening. There were two main kinds of alveolar flow fields, vortex and radial flows. As the acinar generation number increased, the vortex of the alveolar flow was gradually squeezed to the proximal alveolar wall from the alveolar center. The 22nd generation was the last generation where the vortex and radial flows coexisted (Figure 4G), which was different from the alveolar flow patterns with the horizontally placed chip [28]. Only at the 23rd acinar generation did the vortex flow completely disappear (Figure 4H). Gravity had a weak influence on the alveolar flow pattern and it enhanced the influence of the vortex flow at the last several acinar generations.

#### 3.2.2. Particle Tracking in Alveoli at Different Generations

Particle transport varied at different acinar generations because of different alveolar flow patterns, and particle transport also varied at different locations of an alveolus. Particles with a diameter of 5 μm were chosen as the substitutes in the mixture solution for the typical particles with a diameter of 0.5 μm in the alveolar air. It is worth noting that, although the alveolar geometry at the 23rd generation was used to represent those at other generations for the specific reason of ease in the microfabrication process, the dimensionless flow parameters listed in Equations (1)–(5) were precisely matched for each generation to guarantee the dynamic similarity.

**Figure 5:**
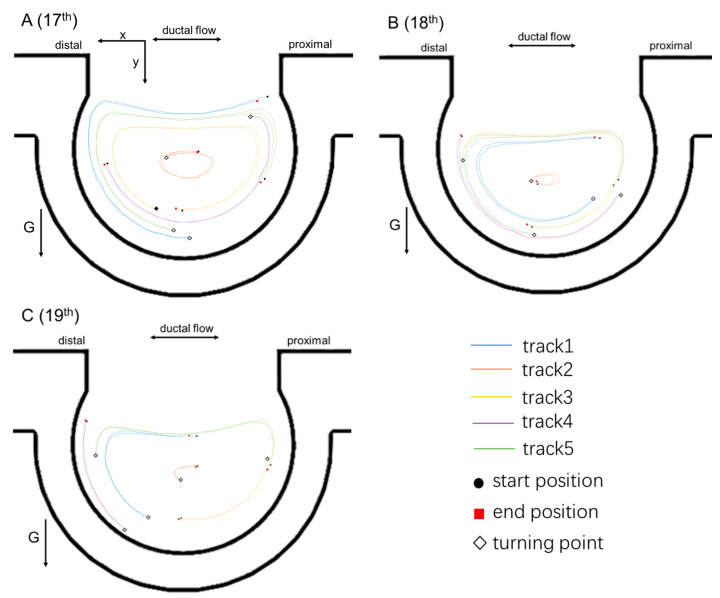

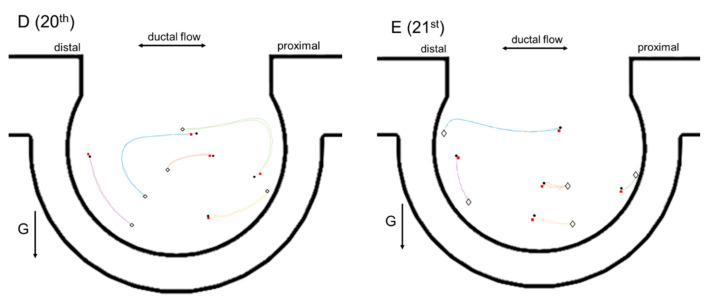
Particle trajectories at different acinar generations over one breathing cycle. (**A**–**E**) are particle tracks inside the alveoli at the 17th to 21st acinar generations, respectively.

The 5 μm silver particles at five typical locations (upper, middle, lower, left, and right inside the vertically placed alveolar chip) of the alveolus at the 17th to 21st generations were tracked over one breathing cycle, as shown in Figure 5. Lines with different colors were used to differentiate between individual particle trajectories, and tracks 1–5 represent particle trajectories at five typical locations, respectively. (1) At the 17th to 21st acinar generations (Figure 5A–E), the particles inside the alveolar chip could not return to the start position after one breathing cycle, and the particle trajectories in the exhalation were similar to those in the inhalation, but they did not overlap exactly. During the inhalation, the particles inside the alveolus were transported in a counterclockwise direction, which was closely related to the alveolar flow patterns. (2) For particles at the five typical locations at different acinar generations, the positions of the tracked particles at the center of the alveolus (track 2) changed more slowly, and the positions of particles at the alveolar opening (track 1) changed more quickly. This was related to the velocity magnitudes of different places in the alveolar flow field. (3) The distance between the end position and the start position became larger for particles in different places as the acinar generation number increased.

**Figure 6:**
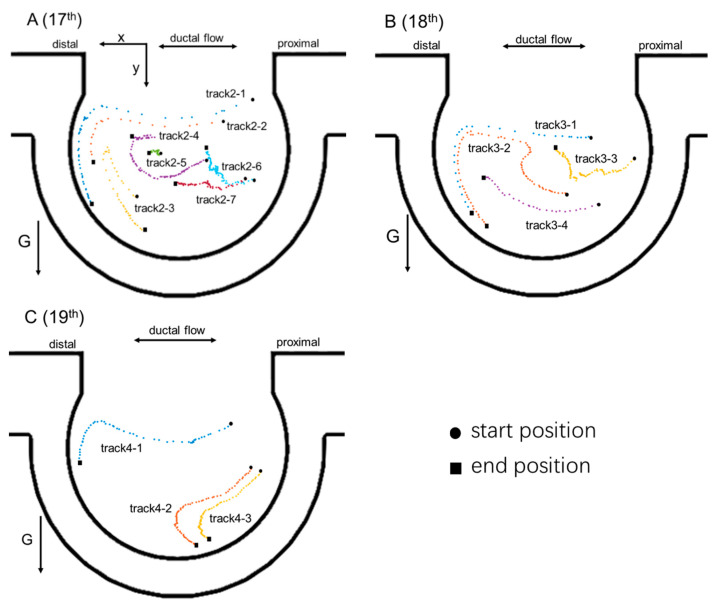

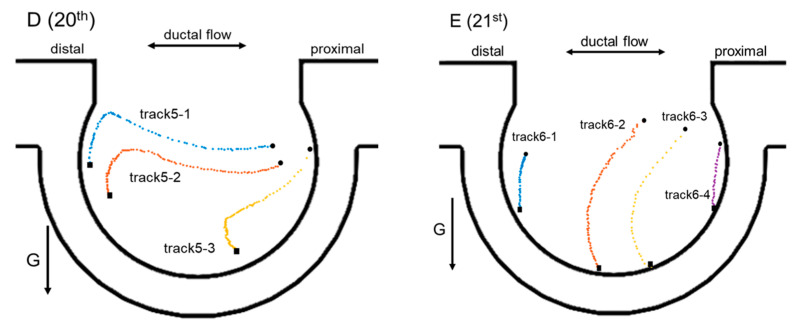
Particle positions at end of each cycle at different acinar generations. (**A**–**E**) are particle tracks inside the alveoli at the 17th to 21st acinar generations, respectively. Colors have no significance other than to differentiate between individual particles.

Because the microparticles transported in the alveoli followed the streamlines closely, there was no obvious difference between the particle trajectories in different places. A healthy adult takes tens of thousands of breaths per day. In order to investigate the particle transport and sedimentation in the alveoli at different generations, it was necessary to track and analyze particle transport in the alveoli for several breathing cycles. Figure 6 shows the 5 μm silver particle positions at end of each breathing cycle from the 17th to 21st acinar generations. The dots with different colors indicate particle positions at different times, and the adjacent dots with the same color differ in time by one breathing cycle. The *x*-axis is in line with the direction of the alveolar duct, and the *y*-axis is in line with the gravitational direction. With the increase in breathing cycles, the particles moved further from their start positions.

(1) The intensity of the vortex flow inside the alveolus and the shear flow in the alveolar duct directly affected the particle transport in the alveoli. At the 17th to 18th acinar generations, the intensity of the vortex flow was so high that the particle end positions at each breathing cycle formed vortex trajectories. It was almost impossible for the particles inside the vortex to escape from the vortex (track 2–4, track 2–6, track 3–2, and track 3–3), and it was difficult for the particles outside the vortex to enter the vortex (track 2–5). The particles track 2–3 and track 2–5 transported around the vortex in the early breathing cycles, and the directions of particle transport reversed after a certain number of breathing cycles where the shear flow should have had a greater effect on the particle transport than the vortex flow. The phenomenon also occurred when the particle track 3-2 moved a certain distance from the alveolar opening. (2) With the increase in breathing cycles, the particles (track 2–1, track 3–1, track 4–1, and track 5–1) at the alveolar opening all moved from the proximal alveolar wall to the distal alveolar wall, and they all had the tendency to deposit on the distal alveolar wall. At the earlier generations, most particles moving into the alveoli from the duct had the tendency to deposit on the distal alveolar wall. At the 21st acinar generation, the vortex flow inside the alveolus and the shear flow in the duct had a smaller impact on particle transport, and the particles at different locations of the alveolus almost directly settled to the bottom from their start positions. In summary, the particles tended to deposit on the distal alveolar wall at the early generations (from 17th to 20th), which is consistent with the simulated results of high deposition densities near the alveolar entrance ring, obtained by Tsuda et al. [8] and Haber et al. [9]. However, we also observed that most particles directly settled to the bottom of the alveolus at the last several generations from the 21st generation. We could also find some hints of the irreversibility and gravity effect on the particle transport and deposition from the studies performed by Fishler et al. [22] and Dong et al. [31], showing the trajectory within a limited number of cycles.

### 3.3. Effects of Particle Size on Sedimentation

**Figure 7:**
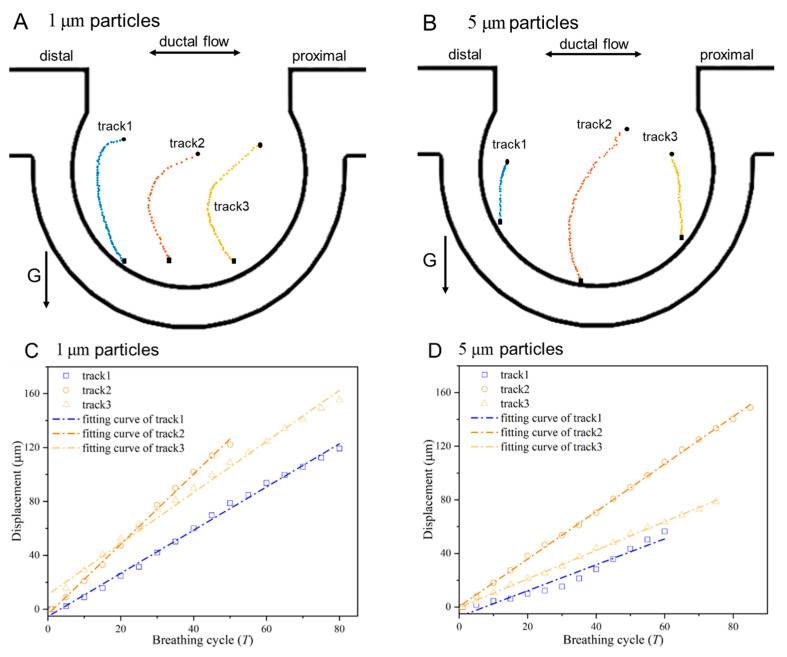

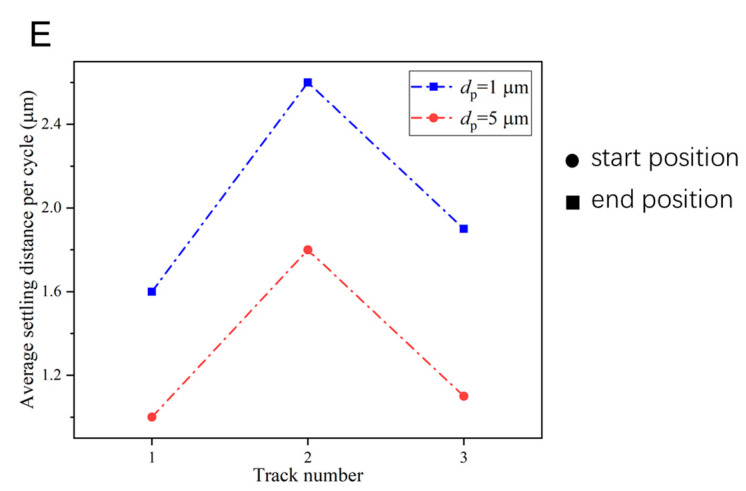
Comparison of settling rates of particles of different sizes in the alveolar chip of the 21st generation. (**A**) Tracked positions of 1 μm particles at end of each breathing cycle; (**B**) tracked positions of 5 μm particles at end of each breathing cycle; (**C**) 1 μm particles settling displacements along the gravitational direction; (**D**) 5 μm particles settling displacements along the gravitational direction; (**E**) average settling distance per breathing cycle of different size particles.

The particle size had an important influence on the settling rate in the alveoli. The settling process of particles of two sizes in the alveoli was investigated in this paper. Figure 7 shows a comparison of the settling rates of silver particles with diameters of 1 μm and 5 μm in the vertically placed alveolar chip at the 21st acinar generation. The reason for selecting the 21st generation to study the size effect on particle sedimentation was that the particles start to directly settle to the bottom from the 21st generation, as shown in Figure 6, and we wanted to work out what different phenomena could be observed for particles of different sizes. Figure 7A,B show the end positions of each breathing cycle of the particles in the middle and on both sides of the alveolus. Colors have no significance other than to differentiate between individual particles, and the particle start positions were all at the alveolar opening. The trajectories of particles with a diameter of 1 μm had a greater degree of curvature in the middle of the alveoli than that of 5 μm particles, and it revealed that the drag force had a greater effect on the movement of 1 μm particles compared to 5 μm particles. All the particles would eventually deposit on the bottom of the alveolus. Figure 7C,D show the settling distance along the gravitational direction over the breathing cycle of the particles in Figure 7A,B, respectively. The particle displacements all increased linearly with the breathing cycles. The fitting degrees of track 1–3 in Figure 7C were 0.996, 0.997,0.989, respectively, and the fitting degrees of track 1–3 in Figure 7D were 0.949, 0.999,0.998, respectively. The slopes of these linear fits were used as average settling distances per breathing cycle of corresponding particles, as shown in Figure 7E. Particles (track 2) in the middle of the alveolus settled faster than those (track 1 and track 3) on either side at the same generation. In addition, the smaller particles settled faster than the larger ones in the same area at the 21st acinar generation. The particle transport and sedimentation in the alveoli was mainly affected by the drag force. The drag force played a leading role in particle transport and sedimentation in the alveoli, and the trajectories of particle positions at end of each breathing cycle were similar to the streamlines.

## 4. Conclusions

The detailed microparticle behavior in the human alveoli of different generations has been experimentally investigated in this study. Taking advantage of the microfluidic technique, a dynamic respiration process with geometry and kinematic similarity was built in an alveolar chip. It was found that particles inside the alveoli either escape from the inlet side of the alveolar duct or stay in the alveoli after sufficient breathing cycles. It revealed the irreversibility of particle transport in the alveoli. Particles in the alveoli of different generations were tracked for a sufficient number of breathing cycles. The vortex flow inside the alveoli and the shear flow in the duct acted together on the particle behavior in the alveoli. The intensity of shear flow had an effect on the locations of particle deposition. At the earlier generations, most particles moving into the alveoli from the duct would deposit on the distal alveolar wall. At the last several generations, there were no particular deposition areas for particles in the alveoli. Comparison of settling rates between particles of different sizes indicated that the smaller particles settle faster, which revealed that the convective flow was the main factor affecting the particle transport and deposition. The observed particle transport and sedimentation laws in the alveoli provide not only straightforward evidence to verify previous theoretical predictions, but also significant data for disease diagnosis and drug delivery.

## Data Availability

Not applicable.
